# Elucidating the Possible Involvement of Maize Aquaporins in the Plant Boron Transport and Homeostasis Mediated by *Rhizophagus irregularis* under Drought Stress Conditions

**DOI:** 10.3390/ijms21051748

**Published:** 2020-03-04

**Authors:** Gabriela Quiroga, Gorka Erice, Ricardo Aroca, Juan Manuel Ruiz-Lozano

**Affiliations:** Departamento de Microbiología del Suelo y Sistemas Simbióticos, Estación Experimental del Zaidín (CSIC), Profesor Albareda nº 1, 18008 Granada, Spain

**Keywords:** arbuscular mycorrhizal symbiosis, aquaporins, boron mobilization, water deficit

## Abstract

Boron (B) is an essential micronutrient for higher plants, having structural roles in primary cell walls, but also other functions in cell division, membrane integrity, pollen germination or metabolism. Both high and low B levels negatively impact crop performance. Thus, plants need to maintain B concentration in their tissues within a narrow range by regulating transport processes. Both active transport and protein-facilitated diffusion through aquaporins have been demonstrated. This study aimed at elucidating the possible involvement of some plant aquaporins, which can potentially transport B and are regulated by the arbuscular mycorrhizal (AM) symbiosis in the plant B homeostasis. Thus, AM and non-AM plants were cultivated under 0, 25 or 100 μM B in the growing medium and subjected or not subjected to drought stress. The accumulation of B in plant tissues and the regulation of plant aquaporins and other B transporters were analyzed. The benefits of AM inoculation on plant growth (especially under drought stress) were similar under the three B concentrations assayed. The tissue B accumulation increased with B availability in the growing medium, especially under drought stress conditions. Several maize aquaporins were regulated under low or high B concentrations, mainly in non-AM plants. However, the general down-regulation of aquaporins and B transporters in AM plants suggests that, when the mycorrhizal fungus is present, other mechanisms contribute to B homeostasis, probably related to the enhancement of water transport, which would concomitantly increase the passive transport of this micronutrient.

## 1. Introduction

In higher plants, the metalloid boron (B) is an essential micronutrient, mainly because of its functional and structural roles in primary cell walls, where it crosslinks the pectin polysaccharide rhamnogalacturonan-II (RG-II) to form a network [1,2]. Nevertheless, B may have other, different functions in cell division and elongation, membrane integrity, pollen tube growth and germination [3] or phenolic and nitrogen metabolism [4]. Besides its main role in plant physiology, both high and low B levels can negatively impact crop yield and quality. Thus, plants need to maintain B concentration in their tissues within a narrow range by regulating transport processes [5]. Deficiency in B usually occurs in areas with high rainfall, since boric acid is quite soluble and easily leached by rainfall [5]. On the contrary, B toxicity naturally occurs in arid and semiarid soils, but it can also be the consequence of fertilization, irrigation or mining [6]. In Spain, B excess in soils has been attributed to the use of water from desalinating plants and waste treatment [7]. In any case, under sustained drought stress, the decrease in plant transpiration can lead to B deficiency, negatively impacting plant performance.

Boron is taken up by roots as boric acid at physiological pH values, thus, unlike other essential nutrients that are absorbed as ions, it is consumed as an uncharged molecule [8]. Its cell membrane permeability is relatively high, and it was considered for a long time that the B uptake and transport in plant roots was only a passive process. Nonetheless, the presence of active transport and protein-facilitated diffusion for this nutrient was later demonstrated [9,10,11]. Thus, depending on B availability, the transport of B can follow three different molecular pathways: (1) passive diffusion through biological membranes; (2) facilitated transport; (3) active transport. Members of the Nodulin 26-like intrinsic protein (NIP) aquaporin subfamily have been identified as key channel molecules in the uptake and transport of B in roots. Indeed, among the different plant aquaporin subfamilies, NIPs are a versatile group with a high diversity of substrates, including water, glycerol, silicon, urea, ammonia, hydrogen peroxide or boric acid and a broad range of subcellular localization patterns, which highlights their diverse physiological functions [12,13]. In *Arabidopsis thaliana*, *AtNIP5;1* was found to be crucial under B limitation [10]. BOR-1 is a plasma membrane protein identified in *A. thaliana* as a B efflux transporter. It is crucial under B limitation for xylem loading of the nutrient [14]. BOR-like transporters and NIPs are frequently present in the same cells but localized at opposite cell sides, allowing the transcellular flux of B within the plant organs [15]. Functional orthologues of these proteins were found in different crops such as rice, wheat or barley [16,17,18,19]. Moreover, an aquaporin isoform from sugar beet has been recently described with a role in plant B homeostasis and abiotic stress response [20].

Generally, monocots need less B for their normal growth than dicotyledonous species [21] and, in particular, maize was considered to be a low B-demanding cereal. However, B deficiency also affects this crop worldwide [22], especially at the reproductive stage, since this micronutrient is especially important for the adequate development of inflorescences and tassels [23]. This problem can be exacerbated under drought stress, since the reduction in transpiration rate may lead to B deficiency. Indeed, orthologues of the *Arabidopsis* B channels and transporters were found in maize. *ZmNIP3;1* (*TSL1*) was shown to be crucial for B transport within the plant, as well as for its reproductive and vegetative development [24,25]. Moreover, other maize aquaporins—ZmTIP1;1, ZmTIP2;1, ZmNIP1;1 and ZmNIP2;2—were found to transport B in yeast [26]. As an efflux transporter, the *ZmRTE* gene was found to be a functional orthologue of *AtBOR1* [27], and three additional B transporters genes (*ZmRTE2*, *ZmRTE3* and *ZmRTE6*) have been identified in maize [21]. The diversity of B transporters highlights the tight regulation of B homeostasis in maize, which may be even more important under drought stress conditions.

Most crop plants form mutualistic symbiosis between their roots and arbuscular mycorrhizal (AM) fungi. These fungi increase the surface of plant root systems, enhancing water and nutrient uptake and also providing tolerance for biotic and abiotic stresses [28]. In particular, the beneficial effects of AM fungi under drought stress have been widely studied [29,30]. Aquaporins were recognized as important elements in both water and nutrient exchanges during the AM symbiosis [31] and, in line with this, 16 out of 36 aquaporins from maize were found to be differentially regulated by AM symbiosis during drought stress [26]. Given the diversity of substrates that can be transported by these AM-regulated aquaporin isoforms, they may have a role in the regulation of important physiological processes [32], and thus the elucidation of their in planta transport capacities is necessary to better understand the process of AM-induced drought tolerance.

Among the effects of the AM symbiosis in plant performance during drought stress, increased levels of ions are often observed [33]. Although not much information is available about AM symbiosis and B homeostasis in the host plant, a recent work has shown the beneficial effect of this symbiosis decreasing B toxicity in leaves and roots when applied to a citrus rootstock, which consequently increased plant tolerance to this stress [7].

The present study aimed to assess whether B has a role in planta as an aquaporin substrate in the AM-enhancement of plant performance during drought stress. With this purpose, different concentrations of B were applied in the nutrient solution to non-inoculated and mycorrhizal plants that were submitted or not submitted to a water deficit treatment. These B concentrations were chosen on the basis of bibliography for different plant species, including *Zea mays* [18,22,34] and trying to keep B levels within the narrow range of B concentration that avoid B excess and toxicity. We tested three B concentrations, 0 (no B added to the nutrient solution, meaning that the only B available comers from the traces of B in such solution and the growing medium), 25 and 100 μM. The results obtained improve our knowledge about the mechanisms of AM symbiosis in enhancing tolerance to water deficit.

## 2. Results

### 2.1. Plant Biomass and Symbiotic Development

The total plant dry weight was not significantly affected by B levels under well-watered conditions, with the exception of non-AM plants under high B concentrations (B100), which showed a smooth depression of growth. Mycorrhization significantly enhanced plant dry weight under medium and high B concentrations (B25 and B100) compared to non-AM plants at the same B concentrations (Table 1).

Under drought stress, AM plants presented a higher plant dry weight compared to non-AM plants at the three B concentrations assayed (Table 1).

The percentage of root colonization was not affected by the B concentration under well-watered conditions (near 50%). Under water deficit conditions, mycorrhization was slightly increased at B100 as compared to B0 plants (Table 1).

### 2.2. Stomatal Conductance (gs)

Mycorrhization positively affected stomatal conductance when the plants were well watered, despite the different B concentrations. However, the opposite effect occurred under drought stress, where mycorrhization decreased gs at B0 and B25. At B100, nonetheless, the differences were not significant (Table 1).

### 2.3. Chlorophyll Content and Efficiency of Photosystem II

Chlorophyll content, measured with SPAD, did not show differences due to mycorrhization or B levels under well-watered conditions, with the exception of AM plants at B100, which slightly increased chlorophyll levels. In the case of drought stress, mycorrhization increased chlorophyll levels under all B concentrations compared to non-AM plants (Table 1).

The efficiency of photosystem II was not affected by B concentration in non-AM plants, but decreased in AM plants at B100, as compared to non-AM ones. During drought stress, there was no effect of any of the factors on the efficiency of photosystem II (Table 1).

### 2.4. Mineral Content of Roots and Shoots

**Figure 1:**
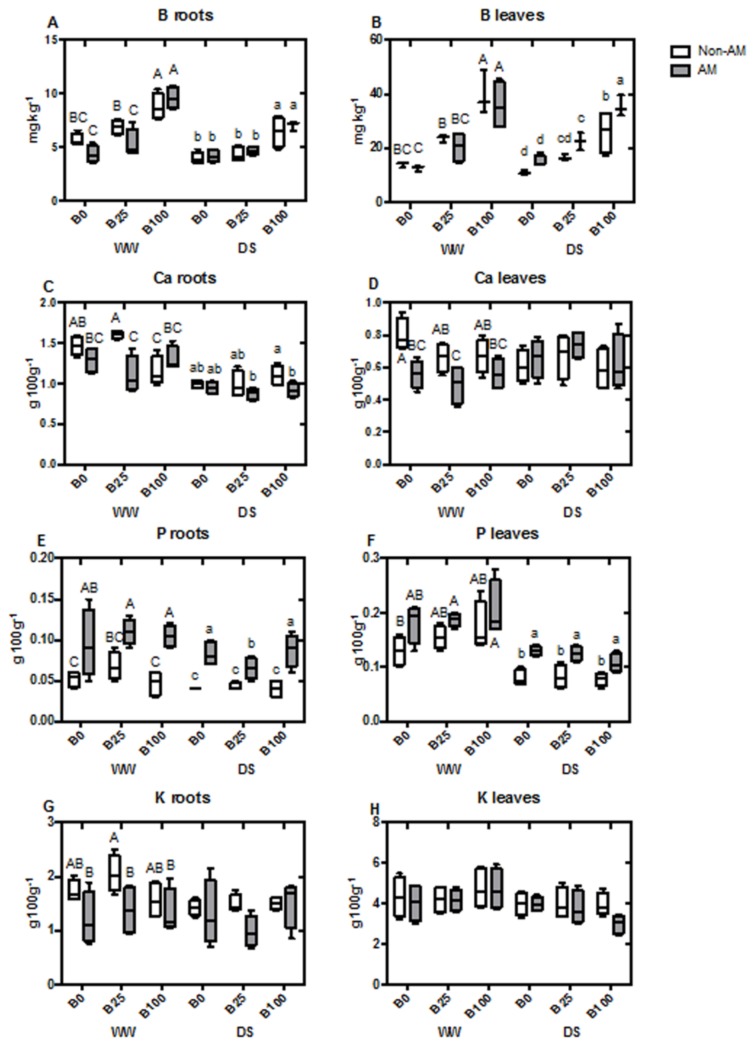
Boxplots representing concentrations of B in roots (**A**) and in leaves (**B**), Ca in roots (**C**) and in leaves (**D**), P in roots (**E**) and in leaves (**F**) and K in roots (**G**) and in leaves (**H**). Boxes represent the interquartile range, with the line representing the median, whiskers represent maxima and minima within 1.5 times the interquartile range. Different letters indicate significant differences among treatments (*p* < 0.05) based on Duncan’s test.

**Figure 2:**
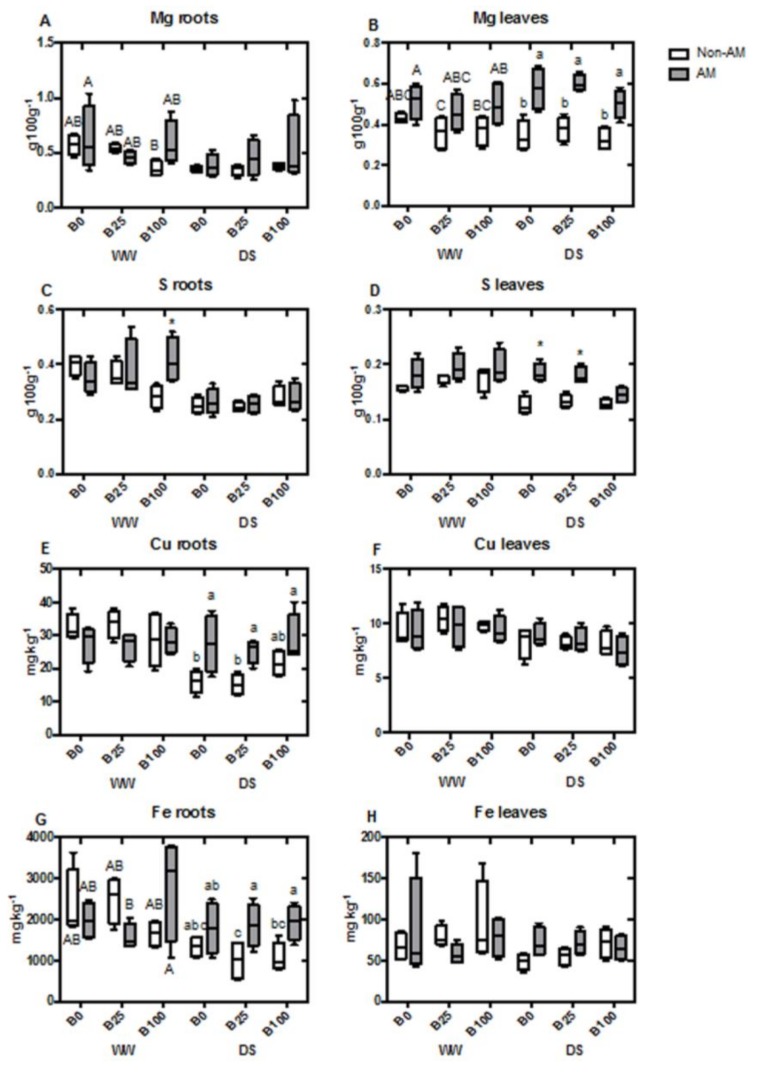
Boxplots representing root and leaf concentrations of Mg in roots (**A**) and in leaves (**B**), S in roots (**C**) and in leaves (**D**), Cu in roots (**E**) and in leaves (**F**)and Fe in roots (**G**) and in leaves (**H**). Boxes represent the interquartile range, with the line representing the median, whiskers represent maxima and minima within 1.5 times the interquartile range. Different letters indicate significant differences among treatments (*p* < 0.05) based on Duncan’s test. Asterisks indicate significant differences between non-AM and AM plants within each treatment, according to *t*-test.

**Figure 3:**
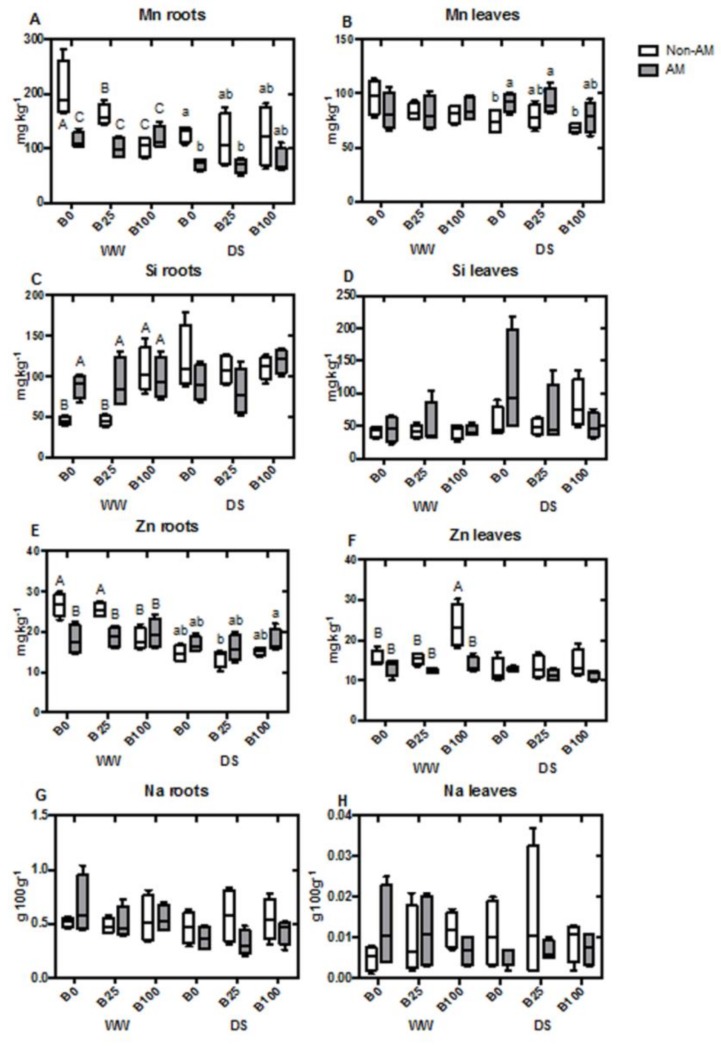
Boxplots representing root and leaf concentrations of Mn in roots (**A**) and in leaves (**B**), Si in roots (**C**) and in leaves (**D**), Zn in roots (**E**) and in leaves (**F**)and Na in roots (**G**) and in leaves (**H**). Boxes represent the interquartile range, with the line representing the median, whiskers represent maxima and minima within 1.5 times the interquartile range. Different letters indicate significant differences among treatments (*p* < 0.05) based on Duncan’s test.

Boron concentration in roots increased in plants irrigated with B100 in both water treatments, regardless of AM fungal inoculation. However, B25 did not significantly increase root B concentration compared to plants that did not receive B (Figure 1A). In leaves, the same trend was observed under well-watered conditions. However, under water deficit, AM plants slightly increased B levels at B100 compared to non-AM plants, although both plant groups presented a higher concentration when compared with the other B treatments (Figure 1B). Ca concentration was significantly decreased due to mycorrhization in well-watered plants, only at B25. Under drought, however, this decrease was significant in AM plants at B100 (Figure 1C). In leaves, the same drop in Ca concentration occurred in well-watered AM plants at B0 and B25, but no significant differences were detected at B100 or during drought stress (Figure 1D). P concentration in roots was increased by AM presence under all B concentrations and water regimes (Figure 1E). In leaves, P concentration only increased significantly with mycorrhization under water deficit conditions, being unaffected by B levels (Figure 1F). Interestingly, K concentration in roots presented a similar trend as Ca accumulation and was decreased in AM plants at B25 under well-watered treatment. K concentration didn’t vary under drought stress or in leaves with any of the treatments (Figure 1 G,H). Mg concentration in roots did not show significant differences due to AM inoculation, B concentration or water regime (Figure 2A). However, in leaves it was increased by mycorrhization under all the different conditions (although this was not significant under well-watered conditions) (Figure 2B). S concentration presented a similar trend to Mg. In roots, the changes in S concentration were not significant (Figure 2C), but the concentration of this compound in leaves generally increased with mycorrhization, while it was only significant at B0 or B25 during drought stress treatment (Figure 2D). Cu content in roots was regulated by AM during drought stress, increasing it level to achieve almost the concentrations of well-watered plants (Figure 2E). Nonetheless, no changes were observed in the leaves of the different treatments (Figure 2F). Concentration of Fe in roots was generally high, but significantly enhanced in the AM roots of droughted plants at B25 and B100 (Figure 2G). Once again, the concentration of this element in leaves was not affected by the different treatments (Figure 2H). In the case of Mn, root concentration decreased with mycorrhization under well-watered conditions at B0 and B25, not being affected by AM at B100. Under drought stress, a significant decrease in Mn concentration with AM inoculation was only observed at B0 (Figure 3A). In leaves, the opposite effect was observed during drought at B0, with AM increasing the concentration of this nutrient (Figure 3B). Si concentration in roots was increased by AM at B0 and B25 under well-watered conditions, while, at B100, levels of non-AM plants were higher and similar to those of AM-plants. Under water deficit, no differences were observed (Figure 3C), as well as in leaves (Figure 3D). Zn content in roots decreased in well-watered AM-plants at B0 and B25. At B100, levels of Zn decreased in both non-AM and AM plants. Under drought, no significant differences were detected (Figure 3E). In leaves, Zn concentration increased in non-AM plants at B100 during WW conditions (Figure 3F). Na concentration was not affected in either roots or leaves by any of the applied treatments (Figure 3G,H).

### 2.5. mRNA Relative Transcript Abundance of Aquaporins

**Figure 4:**
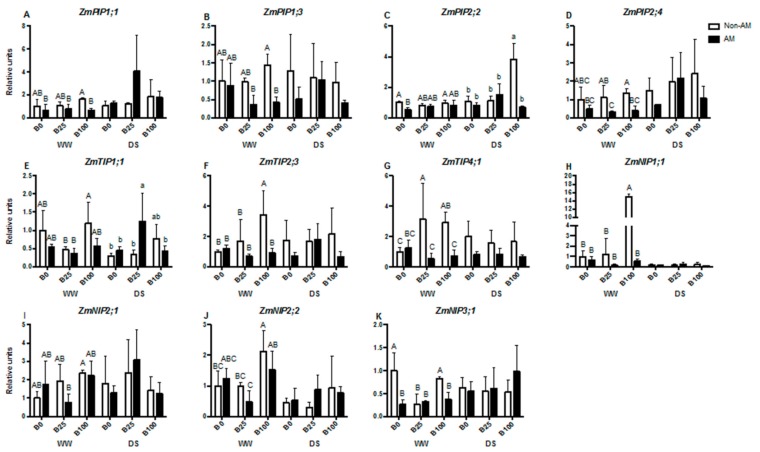
Relative mRNA levels of *ZmPIP1;1* (**A**), *ZmPIP1;3* (**B**), *ZmPIP2;2* (**C**), *ZmPIP2;4* (**D**), *ZmTIP1;1* (**E**), *ZmTIP2;3* (**F**), *ZmTIP4;1* (**G**), *ZmNIP1;1* (**H**), *ZmNIP2;1* (**I**), *ZmNIP2;2* (**J**) and *ZmNIP3;1* (**K**), normalized to *ZmEF1* gene. Plants were inoculated or not inoculated with the AM fungus *R. irregularis*, grown under different B concentrations (0, 25 or 100 μM B) and submitted to two water regimes (well-watered [WW] or drought stress [DS]). Data indicate the mean ± SE for three biological replicates. Different letters indicate significant differences between treatments (*p* < 0.05) based on Duncan’s test.

**Figure 5:**
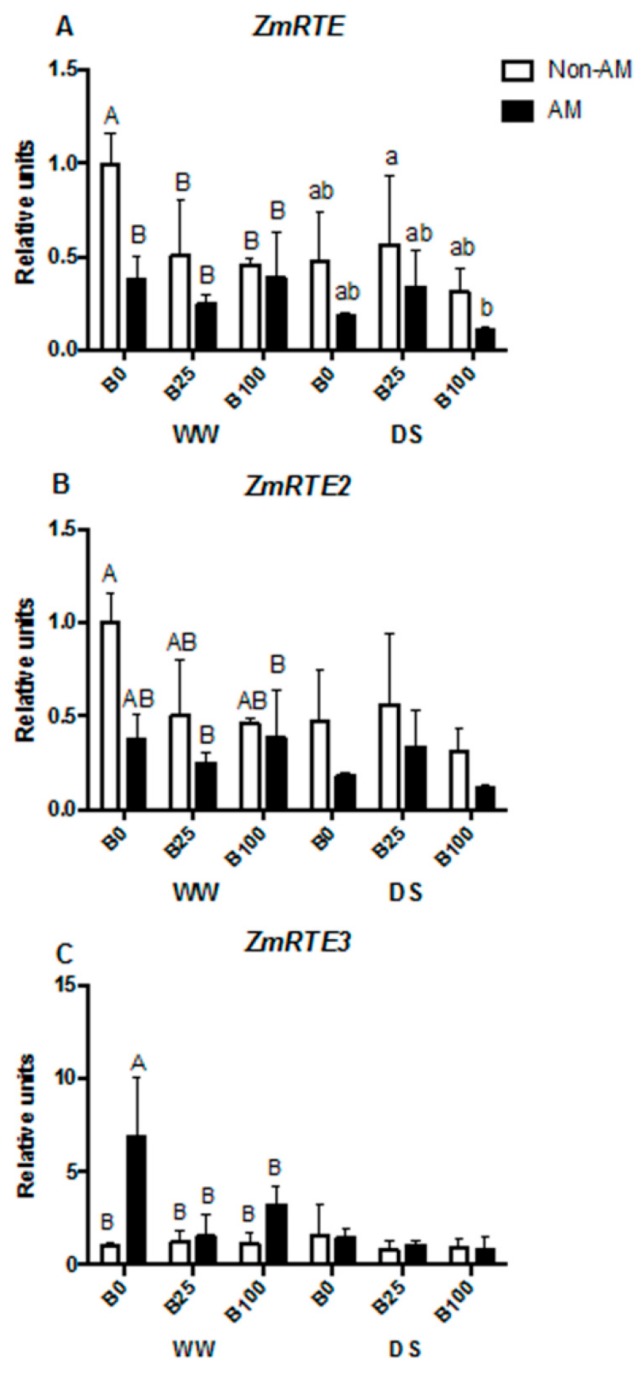
Relative mRNA levels of *ZmRTE* (**A**)*, ZmRTE2* (**B**) and *ZmRTE3* (**C**) normalized to *ZmEF1* gene. Plants were inoculated or not with the AM fungus *R. irregularis*, grown under different B concentrations (0, 25 or 100 μM B) and submitted to two water regimes (well-watered [WW] or drought stress [DS]). Data indicate the mean ± SE for three biological replicates. Different letters indicate significant differences between treatments (*p* < 0.05) based on Duncan’s test.

Eight plant aquaporins selected in previous studies due to their AM regulation [35] were analyzed. Three additional aquaporins that potentially transport B, *ZmNIP1;1*, *ZmNIP2;2* and *ZmNIP3;1* were also studied. Moreover, three B efflux transporters, *ZmRTE*, *ZmRTE2* and *ZmRTE3* [21], were also included in this study. *ZmPIP1;1* and *ZmPIP1;3* mRNA levels were not regulated by B under full irrigation conditions, although AM plants decreased transcript levels compared to non-AM ones at B100. Under water deficit conditions, no differences were observed among treatments (Figure 4A,B). In the case of *ZmPIP2;2*, a down-regulation due to mycorrhization was evident at B0 in well-watered plants. On the contrary, an important up-regulation of this gene occurred in non-AM plants at B100, increasing mRNA levels four times compared to B0 plants (Figure 4C). *ZmPIP2;4* transcript levels decreased with mycorrhization under well-watered conditions, although the effect was only significant at B25 and B100. However, this effect was not observed under drought stress (Figure 4D). No significant changes were observed in *ZmTIP1;1* transcript abundance in well-watered plants with any of the applied treatments. However, during drought stress, mycorrhizal plants up-regulated this gene at B25 compared to non-AM plants and to the other B concentrations (Figure 4E). *ZmTIP2;3* mRNA levels increased at B100 in non-AM plants under well-watered conditions. However, under drought stress conditions, no significant differences in gene expression were found (Figure 4F). Generally, AM plants decreased *ZmTIP4;1* transcript abundance, although the effect was only significant for well-watered plants at B25 or B100. These plants also have higher levels of ZmTIP4;1 transcripts than plants at B0 (Figure 4G). In the case of *ZmNIP1;1*, a strong up-regulation occurred in well-watered non-AM plants at B100, but no significant differences were observed under drought stress (Figure 2H). No significant differences in transcript accumulation were observed for *ZmNIP2;1* gene (Figure 4I). *ZmNIP2;2* transcript abundance slightly increased, with high B (B100) in both AM and non-AM plants under well-watered conditions. Drought did not significantly affect the expression of this gene (Figure 4J). Mycorrhization decreased *ZmNIP3;1* expression of well-watered plants at B0 and B100, while no significant effect was observed under drought stress (Figure 4K). Interestingly, *ZmRTE* was generally down-regulated in all treatments compared to non-AM plants at B0, and this result was similar for *ZmRTE2*, although only significant in well-watered AM plants at B25 and B100 (Figure 5A,B). In the case of *ZmRTE3*, however, a significant up-regulation occurred with mycorrhization at B0 under well-watered conditions (Figure 5C).

### 2.6. Aquaporin Protein Accumulation and PIP2s Phosphorylation Status

**Figure 6:**
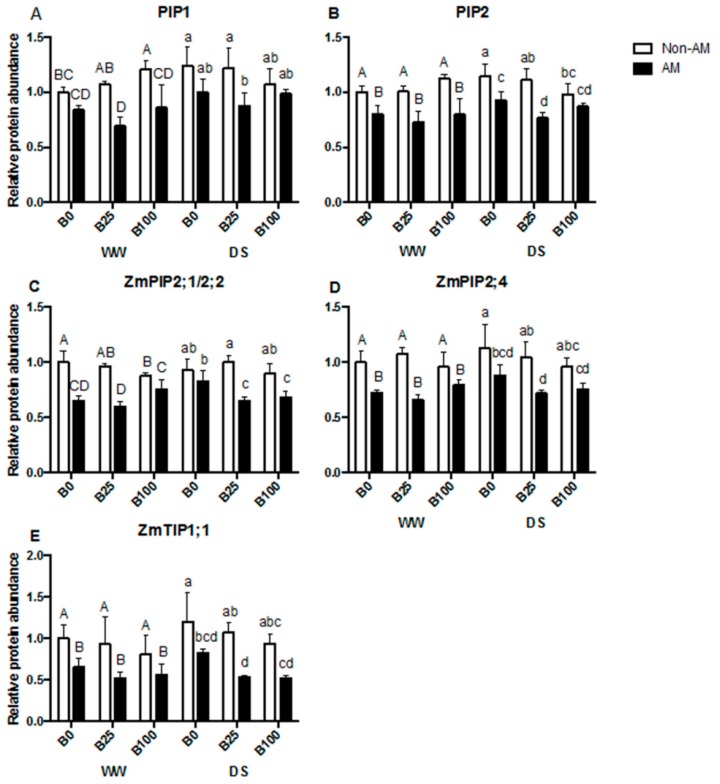
Relative protein abundance for PIP1 (**A**), PIP2 (**B**), ZmPIP2;1/2;2 (**C**), ZmPIP2;4 (**D**) and ZmTIP1;1 (**E**) in the microsomal fraction of roots from plants inoculated or not with the AM fungus *R. irregularis*, grown under different B concentrations (0, 25 or 100 μM B) and submitted to two water regimes (well-watered [WW] or drought stress [DS]). Data indicate the mean ± SE for three biological replicates. Different letters indicate significant differences between treatments (*p* < 0.05) based on Duncan’s test.

A general decrease in PIP accumulation was observed in AM plants with all the analyzed antibodies: the general anti-PIP1 and PIP2 and the isoform specific anti-ZmPIP2;1/2;2, antiPIP2;4 and anti-TIP1;1. This effect was significant regardless of the water or the B treatment. The different B concentrations did not impact protein accumulation (Figure 6A–E).

**Figure 7:**
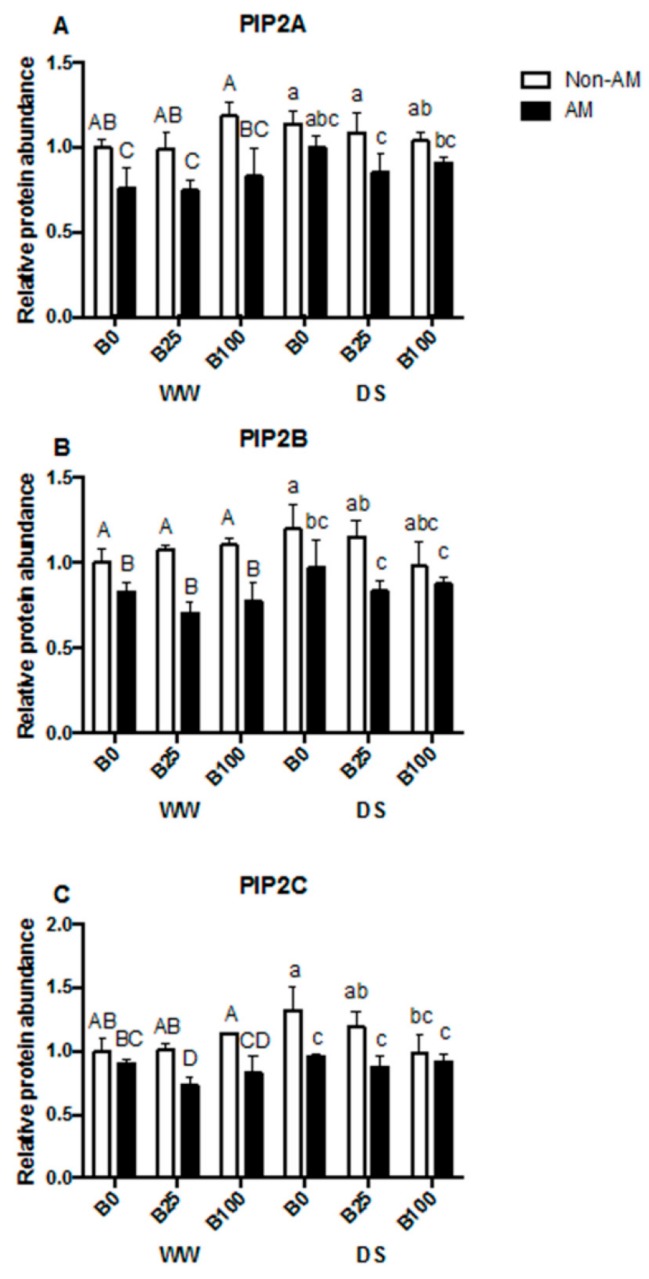
(**A**) PIP2A (Ph-Ser280), (**B**) PIP2B (Ph-Ser283) and (**C**) PIP2C (PhSer280/Ser283) relative protein abundance in the microsomal fraction of roots from plants inoculated or not with the AM fungus *R. irregularis*, grown under different B concentrations (0, 25 or 100 μM B) and submitted to two water regimes (well-watered [WW] or drought stress [DS]). Data indicate the mean ± SE for three biological replicates. Different letters indicate significant differences between treatments (*p* < 0.05) based on Duncan’s test.

The phosphorylation of PIP2 proteins in different serine residues (PIP2A-Ser 280, PIP2B-Ser 283 and PIP2C-Ser 280/283) was also generally decreased by mycorrhization, but not affected by B concentration or drought stress treatment (Figure 7A–C).

## 3. Discussion

The present study aimed to understand whether maize aquaporins regulated by the AM symbiosis are involved in the B transport and homeostasis in planta under water deficit conditions. There is not much information available about the role of AM symbiosis in plant B homeostasis, and, as far as we know, this is the first study dealing with this topic in AM maize plants under drought stress.

At the physiological level, no detrimental effects of either low or high B concentrations were observed in this experiment, under well-watered or drought stress conditions (Table 1). This is not surprising, since the B levels assayed were chosen in the range of concentrations that avoid deficiency or toxicity levels [21,36]. The benefits of AM inoculation (especially under drought stress) were similar under the range of B concentrations assayed (as evidenced by plant dry weight or chlorophyll contents). As explained above, the apparent lack of physiological response from the plant may be due to the low B requirement of maize, as a monocot species [21]. In fact, during vegetative stages, monocots rarely develop deficiency symptoms [37]. Different B requirements between dicots and monocots may be related to the different composition of their cell walls [38]. In addition, the tolerance of each species to B deficiency or excess is highly variety-dependent [6,39,40], and some low-demanding cultivars may increase B use efficiency, which allows them to develop with a limited amount of this nutrient [40]. Recently, it has been also suggested that B is neither a beneficial nor an essential element for plant growth. Indeed, it was hypothesized that it is maintained in a homeostatic balance within the plant, thanks to the natural selection of constitutive biochemical mechanisms [41].

When B concentrations are not deficient, it moves in the plant during the active transpiration, accumulating where water is lost through stomata in the leaf [42,43]. Moreover, even under non-optimal soil B conditions, transpiration stream was found to be a significant source of B for maize plants [36]. Therefore, in this study it is not surprising that a higher concentration of B was observed in leaves (ranging from 20 to 50 mg kg^−1^) compared to roots (ranging from 4 to 10 mg kg^−1^) (Figure 1A,B) regardless of the B concentration applied. In fact, even under excess of B, roots generally do not reflect any visible symptoms, and B concentrations remain relatively low compared to leaves [6]. In general, mycorrhization did not have an effect on tissue B concentrations, although, during drought, B concentrations in leaves slightly increased with mycorrhizal presence, being only significant at B100 (Figure 1B). As explained earlier, the observed effect may be due to the enhancement of water transport in these plants. In relation to this, ectomycorrhizal fungi were found to enhance B uptake in *Betula pendula*, but the effect was mild and dependent on fungal species [44]. In contrast, mycorrhization decreased uptake of B and concentrations in wheat plants under both, with and without B supply [45]. In a recent study with citrus rootstocks and *R. irregularis*, the symbiosis decreased the toxicity of high B application, accumulating less B in leaves [7]. The disparity in results obtained in different studies suggests that the effect of mycorrhization on plant B homeostasis is very dependent on the plant–fungal combination, as well as on the specific conditions of the experiment. In fact, in the case of micronutrients that are heavy metals, the effect of the AM symbiosis on plant accumulation depend on whether the nutrients are under deficiency in the soil or whether they are in excess. Thus, AM fungi can increase the uptake of low mobility metal micronutrients when plants grow in soils deficient in these elements. In contrast, AM fungi can alleviate their toxicity in polluted soils. This has led to the hypothesis that AM functions as a ‘buffer’ to regulate the plant concentration of these micronutrients depending on their availability in the soil [46], which may also operate for B.

Generally, mycorrhization enhanced the uptake of nutrients, especially under water deficit, as revealed by the higher levels in roots and/or leaves of P, Mg, S, Cu, Fe or Mn (Figure 1, Figure 2 and Figure 3). This effect is one of the most obvious benefits from AM symbiosis, and it is due to the efficiency of the extra-radical mycelial network in penetrating deeper in soils, extracting water and nutrients even under drought stress. This AM-improvement in nutrient uptake has been extensively reported in numerous plant–fungal combinations [47,48,49]. Nonetheless, concentrations of those nutrients were not affected in this study by the different B conditions.

High B levels produce changes in plant water balance, probably as a mechanism to prevent excessive B accumulation. Thus, PIP aquaporins are probably involved in this process, as recently observed in *Arabidopsis* plants [50]. This statement is in agreement with our results, as *ZmPIP2;2* mRNA levels were upregulated in non-AM plants under high B supply during water stress (Figure 4C), which may represent a way to increase water flow in roots and decrease B excess. In fact, ZmPIP2;2 isoform showed high water transport capacity in *Xenopus laevis* oocytes [26,51] and was found to contribute to root cell water permeability changes in maize protoplasts [52]. However, AM plants under the same conditions decreased *ZmPIP2;2* transcript abundance, which suggests that mycorrhizal plants have other mechanisms for regulation of B excess. For instance, AM plants had enhanced P levels in their tissues, as compared to non-AM plants, and it has been described that the interaction of B with P reduces B-excess toxicity [53,54]. AM plants usually exhibit enhanced antioxidant activities, and this has been related to reduced B-excess toxicity [55,56]. The up-regulation of *ZmTIP2;3* in WW non-AM plants at B100 suggests that this aquaporin may be also involved in B homeostasis under high B concentrations (Figure 4F). In any case, further studies are needed to demonstrate the proposed roles of *ZmPIP2;2* and *ZmTIP2;3*.

NIP aquaporins were found to be crucial for the uptake and transport of B within roots [10]. In maize, *ZmNIP3;1* (*TSL1*), an ortholog of *AtNIP5;1*, has been implicated in the transport of B under B-deficient conditions. It was mainly expressed in inflorescences, although it was also found in other plant tissues such as roots [25]. In agreement with this, *ZmNIP3;1* mRNA levels were higher at B0 in non-AM plants compared to other treatments under well-watered conditions, although levels increased again at B100 also in non-AM plants (Figure 4K). However, differential transcriptional regulation of this gene was not observed under drought. In the present study, a strong up-regulation of *ZmNIP1;1* transcript abundance occurred at B100 in non-AM plants under well-watered conditions (Figure 4H). Interestingly, this aquaporin was found to transport B when expressed in yeast [26]. The ar/R regions of major intrinsic proteins were analyzed and compared between rice, maize and Arabidopsis, showing that 30 out of 39 OsMIPs and 23 out of 31 ZmMIPs have identical or similar ar/R signatures to their Arabidopsis counterparts [57]. This is the case for OsNIP1;1, ZmNIP1;1 and AtNIP1;1, which possess the signature W V A R in H2, H5, LE1 and LE2 positions of their ar/R region. All belong to the NIP I group, where the physical characteristics of their ar/R region produce a high pore size of about 3.5 Å and make the transport of big molecules such as glycerol or boric acid possible [58]. Moreover, this region reduces the ability to form hydrogen bonds with water molecules, which explains the low water transport capacity for this group of NIPs and the B transport capacity for ZmNIP1;1 [24]. Thus, *ZmNIP1;1* is a good candidate as a B transporter under high B concentrations. NIPs generally present lower transcript levels compared to other aquaporin subfamilies [59] and *ZmNIP1;1* levels were very low during drought in this study (Figure 4H). This may be the reason why no differences in mRNA levels were detected under the different B concentrations.

*ROTTEN EAR* (*RTE*) is a functional homolog of *AtBOR1* and represents the main B efflux transporter in maize, required for vegetative and reproductive development under B deficiency [27]. In this study, we analyzed other two transporters that also contribute to B transport in different tissues, *RTE2* and *RTE3*. [21] showed that the three genes were expressed in all tissues, but *RTE* and *RTE2* were found in roots with identical expression patterns. Our results are in agreement with this, as *RTE* and *RTE2* showed similar expression patterns in roots, and enhanced levels were found under B0, although only in non-AM plants under WW conditions (Figure 5A,B). Under DS, the regulation of the genes was not strong enough to display differences among treatments. Interestingly, *RTE3* was only upregulated in AM plants, and also at B0 under WW conditions (Figure 5C), suggesting that this gene is differently regulated by the AM symbiosis under B deficiency.

The lack of correlation among B concentrations and B transporters analyzed may be due to the existence of additional uncharacterized B transporters in maize, as previously hypothesized in other studies [36]. Moreover, AM symbiosis generally down-regulated aquaporins and *RTE* genes under all B concentrations and drought conditions. However, during drought, the leaf levels of B increased in AM plants. This could mean than the enhancement of water uptake and transport generally found in mycorrhizal plants [35,60] also leads to an enhanced passive B transport in these plants.

Posttranslational modifications of aquaporins may be also involved in the regulation of water flow as a way to regulate passive B transport. Indeed, the activity of aquaporins must be controlled by regulation mechanisms allowing rapid response to environmental changes. Posttranslational modifications are key to achieving such a rapid and reversible regulation [59], and they affect protein stability, catalytic activity, interaction with other proteins or subcellular localization. Phosphorylation and dephosphorylation are considered key mechanisms regulating the gating of aquaporins and, consequently, their activity [61]. The open state is maintained by phosphorylation in different residues. However, it may also be a way to regulate protein trafficking [62,63]. Kinases and phosphatases are involved in this regulation. There have been found more than 70 different sites of phosphorylation in PIPs, TIPs and NIPs, where the loop B and the N- and C- terminal tails of aquaporins are important sites in channel regulation, often involving serine residues [61,64]. Apart from phosphorylation, other posttranslational modifications have been found to modify aquaporin activity, localization and degradation, such as herteromerization, subcellular trafficking, N-terminal modification, deamidation, glycosylation, methylation or ubiquitination, although most of them are not fully understood and need additional research [13,64].

A general drop in AQP protein levels was observed with the presence of the AM fungus (Figure 6). This is in line with previous results under similar conditions [30,52,60]. B concentrations did not affect aquaporin phosphorylation status, and it seems that drought also did not influence phosphorylation (Figure 7A–C). However, mycorrhization decreased phosphorylation levels in the three cases, coinciding again with previous results [60].

In summary, although a range of B concentrations was applied to AM and non-AM plants during well-watered and water deficit conditions, no apparent physiological effect was found in any of the treatments. This result may be due to the low B requirement of maize, or to tolerance related to the specific cultivar. Some aquaporins (*ZmTIP2;3*, *ZmNIP1;1* and *ZmNIP3;1*) and B efflux transporters (*RTE*, *RTE2* and *RTE3*) were regulated under low or high B concentrations, mainly in non-AM plants. In the case of *RTE* genes, the result confirms their previously proposed role in B transport under deficient conditions. In the case of the stated aquaporins, this is the first report investigating a possible role of AM-regulated plant aquaporins in the in planta B transport and homeostasis, although further studies are needed to complete their functional characterization. However, the general down-regulation of aquaporins and B transporters in AM plants suggests that, when the mycorrhizal fungus is present, other mechanisms contribute to B homeostasis, probably more related to the enhancement of water transport, which would concomitantly increase the passive transport of this micronutrient.

## 4. Materials and Methods

### 4.1. Experimental Design

The experiment consisted of a factorial design with three factors: (1) inoculation treatment, with plants inoculated with the AM fungus *Rhizophagus irregularis*, strain EEZ 58 (AM) and non-inoculated control plants (Non-AM); (2) watering treatment, so that half of the plants were subjected to drought stress (DS) for 15 days before harvest while the other half were grown under well-watered (WW) conditions throughout the entire experiment; (3) B treatment, so that plants were irrigated with nutrient solution with three different B concentrations, plants without B in the nutrient solution (B0, only obtaining the B from the very low-soil-containing substrate), plants irrigated with 25 μM of B in the nutrient solution (B25) and plants irrigated with 100 μM of B (B100), resulting in twelve different treatments with six replicates per treatment (*n* = 6), giving a total of 72 plants.

### 4.2. Soil and Biological Materials

The growing substrate consisted of a mixture of soil and sand (1:9 *v*/*v*). The soil was collected at the grounds of IFAPA (Granada, Spain), sieved (2 mm), diluted with quartz-sand (<1 mm) and sterilized by steaming (100 °C for 1 h) on three consecutive days. The undiluted soil had a pH of 8.1 (water); 0.85% organic matter, nutrient concentrations (mg kg^−1^): P, 10 (NaHCO_3_-extractable P); N, 1; K, 110. The soil texture was made of 47.1% silt, 38.3% sand and 14.6% clay.

Seeds of *Zea mays* L. were provided by the Pioneer Hi-Bred (Sevilla, Spain), cultivar PR34B39 that was also used in previous studies [35,65]. Seeds were pre-germinated in sand and then transferred to 1.5 L pots containing 1250 g of the above-described substrate. At planting time, half of the plants were inoculated with ten grams of AM inoculum with *Rhizophagus irregularis* (Schenck and Smith), strain EEZ 58. The inoculum consisted of spores, mycelia, infected root fragments and soil. Non-inoculated plants received a 10 mL aliquot of an inoculum filtrate (<20 μm), in order to provide the natural microbial population present in the inoculum, but free of AM propagules.

### 4.3. Growing Conditions

Plants were grown under greenhouse conditions (average photosynthetic photon flux density 800 µmol m^−2^ s^−1^, 25/20 °C, 16/8 light dark period and 50–60% RH) for a total of eight weeks. Plants were irrigated three times per week with 50 mL of Hoagland nutrient solution [66] modified to contain only 25% of P, in order to avoid the inhibition of AM symbiosis establishment. The Hoagland solution was also modified to provide the different B concentrations (0, 25 and 100μM). Plants received the same amount of water on alternate days. Maize requires 150 g B per Ha for adequate growth [66]. These levels of B were chosen according to the bibliography to grow maize plants in the range of concentrations that avoid deficiency or toxicity levels [21,36]. The drought stress treatment was applied for the last 2 weeks. For that, plants were irrigated with half the water/Hoagland volume of well-watered ones (25 vs. 50 mL). In order to avoid a combination of drought stress plus nutrient deficiency, droughted treatments received 2X Hoagland nutrient solution, so that 25 mL provided the same nutrient levels as 50 mL of the 1X Hoagland nutrient solution used with well-watered plants. This water stress is considered as a severe stress and was similar to that imposed in previous studies [35,65].

### 4.4. Parameters Measured

#### 4.4.1. Biomass Production

The shoot and root system of six replicates per treatment were fresh-weighed at harvest (8 weeks after sowing). Two replicates per treatment were dried in a forced hot-air oven at 70 °C for 2 days and the dry weight (DW) was measured. The determined dry matter content was used to calculate dry weight from the other plant replicates.

#### 4.4.2. Symbiotic Development

Maize roots were stained following the procedure described by [67], in order to visualize and differentiate AM fungal structures. The extent of mycorrhizal colonization was calculated in three replicates per treatment according to the gridline intersect method [68].

#### 4.4.3. Stomatal Conductance

Stomatal conductance (*gs*) was measured with a porometer system (Porometer AP4, Delta-T Devices Ltd., Cambridge, UK), two hours after the onset of photoperiod and following the manufacturer’s recommendations. The second fully expanded youngest leaves from five plants per treatment were used for this measurement. Measurements were taken one day before harvest.

#### 4.4.4. Leaf Chlorophyll Content

Leaf chlorophyll content was estimated four hours after sunrise on the second fully expanded youngest leaf for each plant by using a Chlorophyll Content Measurement System CL-01 (SPAD, Hansatech Instruments ltd., Norfolk, UK). This device determines relative chlorophyll content in leaf samples by measuring dual optical absorbances (620 and 940 nm wavelengths). Relative chlorophyll content was measured in five different plants per treatment one day before harvest.

#### 4.4.5. Photosynthetic Efficiency

The efficiency of photosystem II was measured one day before harvest in light-adapted maize leaves. We used a Fluor-Pen FP100 (Photon Systems Instruments, Brno, Czech Republic), as described previously in [35,52,65], using the second fully expanded youngest leaf of five different plants per treatment.

#### 4.4.6. Mineral Analysis

Analysis of Ca, K, Mg, S and P concentration (g/100g) as well as B, Cu, Fe, Mn, Zn and Si concentration (mg/kg) was determined in four samples (*n* = 4) of shoots and roots of the different treatments by means of inductively coupled plasma-optical emission spectometry (ICP-OES; THERMO ICAP 6000 DUO). The determination was performed by the Ionomic service of the CEBAS-CSIC institute of Murcia, Spain.

#### 4.4.7. RT-qPCR

Total RNA was extracted from maize roots in three biological replicates, as described previously [35]. First-strand cDNA was synthesized with the Maxima H Minus first strand cDNA synthesis kit (Thermo Scientific ^TM,^ Waltham, MA, USA) using 1 µg of purified total RNA, according to the manufacturer’s instructions.

The expression of eight previously selected maize aquaporins [34], plus the aquaporin genes *ZmNIP1;1*, *ZmNIP2;2*, *ZmNIP3;1* and the B transporters-encoding genes *ZmRTE*, *ZmRTE2* and *ZmRTE3* was measured by qRT-PCR using 1 µL of diluted cDNA (1:9) and PowerUp^TM^ SYBR^TM^ Green Master Mix in a QuantStudio^TM^ 3 system (Thermo Fisher Scientific, Waltham, MA, USA). The reaction was carried out at an annealing temperature of 58 °C for all primers and repeated for 40 cycles. For normalization of gene expression values, four reference genes were measured in all the treatments. These genes were tubulin (gi:450292), poliubiquitin (gi:248338), elongation factor 1 (gi:2282583) and GAPDH (gi:22237) [26]. “NormFinder” algorithm [69] (https://moma.dk/normfinder-software) was used to choose the best-performing of these reference gene under our specific conditions. Thus, expression levels were normalized according to the elongation factor 1 (gi:2282583). The relative abundance of transcripts was calculated using the 2^-ΔΔCt^ method [70]. Three biological replicates were used per treatment and the threshold cycle (Ct) of each biological sample was determined in duplicate. Negative controls without cDNA were used in all PCR reactions.

#### 4.4.8. Aquaporins Abundance and PIP2s Phosphorylation Status

For sub-cellular fractionation, pieces of intact roots were ground with 6 mL of a protein extraction buffer containing 250 mM Sorbitol, 2 mM EDTA, 50 mM Tris-HCl (pH 8), and protease inhibitors, according to [71] with slight modifications. All steps were performed at 4 °C. The homogenate was centrifuged during 10 min at 770 *g* and the supernatant obtained was centrifuged again 10 min at 10,000 *g*. Finally, the subsequent supernatant was centrifuged for 2 h at 14,4000 *g* and the obtained pellet (containing the microsomal fraction) was resuspended in 20 µL of suspension buffer (5 mM KH_2_PO_4_, 3 mM KCl, 330 mM sucrose, pH 7.8) and sonicated twice for 5 s. A Bradford analysis was used to quantify total protein amounts. The abundance of specific proteins was measured by ELISA. A 2 µg aliquot of the microsomal fraction was incubated at 4 °C overnight in carbonate/bicarbonate coating buffer, pH 9.6. Afterwards, proteins were cleaned by 3x 10 min washes with Tween Tris-buffered saline solution (TTBS) and blocked at room temperature with 1% bovine serum albumin (BSA) on TTBS for 1 h. After three more washes with TTBS, proteins were incubated at room temperature for 1 h with 100 µL of the primary antibody (1:1000 in TTBS *v*/*v*).

A total of eight different primary antibodies were used. Two antibodies recognize several PIP1s and PIP2s aquaporins, three antibodies recognize the phosphorylation of PIP2 aquaporins in the C-terminal region: PIP2A (Ser-280), PIP2B (Ser-283) and PIP2C (Ser-280/Ser-283) [72]. Finally, we also used antibodies recognizing ZmPIP2;1/2;2, ZmPIP2;4 and ZmTIP1;1 [71]. As secondary antibody, a goat anti-rabbit IgG coupled to horseradish peroxidase (Sigma-Aldrich Co. Madrid, Spain) was used at dilution 1:10,000.

### 4.5. Statistical Analysis

Statistical analyses were performed in SPSS Statistics (Version 23, IBM Analytics). Data were analyzed by one-way ANOVA. Duncan’s or *t*-Test were used to find out differences between means at α = 0.05.

## Figures and Tables

**Table 1:** Percentage of myocorrhizal root length, plant dry weight (DW), stomatal conductance (gs), chlorophyll SPAD values and photosystem Ⅱefficiency in the light –adapted state (∆Fv/Fm^’^) of maize palnts inoculated or not with the AM fungus *Rhizophagus irregularis,* submitted to two water regimes (well-watered –WW- or drought stress DS) and supplied with three B levels (B0- 0 μM, B25- 25 μM and B 100- 100 μM). Data represents the menas of three values ± Se for gs, SPAD and ∆Fv/Fm^’^. Different letter indicates significant differences between treatments (p < 0.05) based on Duncan’s test.

			Mycorrhization (%)	Plant DW (g plant^−1)^	Gs (mmol H_2_O m^−2^s^−1^)	SPAD	∆Fv/Fm^’^
WW	B0	NON-AM	n.d.	3.98±0.18 AB	40.3±8.05 C	10.6±0.66 B	0.65±0.01A
		AM	54.3 ± 4.48 abc	4.06±0.20 AB	148.4±33.0 A	10.5 ±0.54 B	0.66±0.01 A
	B25	NON-AM	n.d.	3.71±0.11 B	53.8±10.3 C	10.6 ±0.43 B	0.63 ±0.01 AB
		AM	47.7±2.60 bc	4.21±0.13 A	105.4±16.6 AB	10.6 ±0.64 B	0.64 ±0.01 AB
	B100	NON-AM	n.d.	3.13±0.16 C	63.9±6.36 BC	10.7 ±0.55 B	0.66 ±0.01 A
		AM	44.3±1.20 c	4.33±0.18 A	117.6±23.4 A	12.6 ±0.65 A	0.62±0.01 B
DS	B0	NON-AM	n.d.	2.86±0.08 b	44.3 ±4.01 ab	5.66 ±0.53 b	0.52 ±0.04 b
		AM	46.3±2.33 bc	3.49±0.13 a	16.9±3.42 c	8.98 ±0.29 a	0.61 ±0.04 ab
	B25	NON-AM	n.d.	2.72±0.09 b	47.6±10.6 a	5.38 ±0.41 b	0.58±0.04 ab
		AM	58.7±6.77 ab	3.63±0.22 a	25.5±5.11c	8.39 ±0.27 a	0.61±0.03 ab
	B100	NON-AM	n.d.	2.72±0.11 b	30.3±3.96 bc	4.92 ±0.18 b	0.62 ±0.04 ab
		AM	64.7±2.90 a	3.71±0.09 a	27.2±2.35 c	8.97 ±1.02 a	0.63 ±0.02 a
